# Non-farmed fish contribute to greater micronutrient intakes than farmed fish: results from an intra-household survey in rural Bangladesh

**DOI:** 10.1017/S1368980016002615

**Published:** 2016-10-05

**Authors:** Jessica R Bogard, Geoffrey C Marks, Abdullah Mamun, Shakuntala H Thilsted

**Affiliations:** 1 School of Public Health, The University of Queensland, Herston Road, Herston, QLD 4006, Australia; 2 Agriculture Flagship, Commonwealth Scientific and Industrial Research Organisation (CSIRO), St Lucia, QLD, Australia; 3 WorldFish, Jalan Batu Maung, Batu Maung, Penang, Malaysia

**Keywords:** Fish, Capture fisheries, Aquaculture, Animal-source foods, Micronutrients, First 1000 d, Intra-household food consumption

## Abstract

**Objective:**

Fish is the most important animal-source food (ASF) in Bangladesh, produced from capture fisheries (non-farmed) and aquaculture (farmed) sub-sectors. Large differences in micronutrient content of fish species from these sub-sectors exist. The importance of fish in diets of vulnerable groups compared with other ASF; contribution from non-farmed and farmed species to nutrient intakes; and differences in fish consumption among age, gender, wealth groups and geographic regions were analysed, using quantitative intra-household fish consumption data, focusing on the first 1000 d of life.

**Design:**

Two-stage stratified sample.

**Setting:**

Nationally representative of rural Bangladesh.

**Subjects:**

Households (n 5503) and individuals (*n* 24 198).

**Results:**

Fish consumption in poor households was almost half that in wealthiest households; and lower in females than males in all groups, except the wealthiest, and for those aged ≥15 years (*P*<0·01). In infants of complementary feeding age, 56 % did not consume ASF on the survey day, despite 78 % of mothers knowing this was recommended. Non-farmed fish made a larger contribution to Fe, Zn, Ca, vitamin A and vitamin B_12_ intakes than farmed fish (*P*<0·0001).

**Conclusions:**

Policies and programmes aimed to increase fish consumption as a means to improve nutrition in rural Bangladesh should focus on women and young children, and on the poorest households. Aquaculture plays an important role in increasing availability and affordability of fish; however, non-farmed fish species are better placed to contribute to greater micronutrient intakes. This presents an opportunity for aquaculture to contribute to improved nutrition, utilising diverse production technologies and fish species, including small fish.

Fisheries in Bangladesh is a diverse and growing food sector, with scores of species commonly consumed from capture fisheries and large growth in the availability of a few local and imported species from aquaculture – the fastest growing food production sector globally^(^ ^1^ ^)^. Bangladesh ranks fifth in fish production from aquaculture globally and the main species farmed are carp, pangasius and tilapia^(^ ^2^ ^,^ ^3^ ^)^. Fish is by far the most important animal-source food (ASF) in diets in Bangladesh^(^ ^3^ ^)^ and is inextricably linked to the culture and livelihoods of the Bangladeshi people. Fish is also widely acknowledged as a nutrient-rich food with high content and bioavailability of micronutrients^(^ ^4^ ^,^ ^5^ ^)^ and as playing an essential role in the diets of vulnerable groups. However, despite its diversity and nutritional importance, our knowledge of consumption of this food group is limited to household-level food acquisition data or a few surveys of small sample size, carried out in very specific geographic locations^(^ ^6^ ^)^. Furthermore, while capture fisheries and aquaculture as production systems are distinct from each other in terms of management and governance, and the foods from these systems (non-farmed and farmed fish) have distinct nutritional profiles^(^ ^4^ ^)^, consumption patterns of these foods are rarely differentiated. Lack of recognition of the complementary roles for capture fisheries and aquaculture to play in contributing to sustainable healthy diets represents an untapped opportunity^(^ ^7^ ^)^.

The ubiquity of malnutrition in all its forms, despite decades of global research, investment and policy making in addressing its immediate determinants, has seen gathering momentum for research which takes a more nuanced approach to understanding dietary quality^(^ ^8^ ^)^. Furthermore, targeting vulnerable groups, particularly women and children during the first 1000 d of life (from conception through to 2 years of age) and women throughout their reproductive years, has been recognised as an essential strategy for prevention of adverse outcomes including poor cognition and stunted growth^(^ ^9^ ^)^. A recent nationally representative survey of rural Bangladesh offers a unique opportunity to apply such approaches to our understanding of diets. In contrast to typical household surveys, this survey includes detailed quantitative 24 h recall data on foods consumed by all household members, including pregnant and lactating women and infants less than 2 years of age. Of particular interest is consumption of nutrient-rich foods, which can provide informative data for designing food-based policies, programmes and interventions to improve diets. Given the importance of fish in diets compared with other ASF, the detailed data on consumption of more than sixty species of fish in this survey are the focus of analysis. Of note is that recent research found large variations in the nutrient composition of small indigenous fish species (SIS), from non-farmed sources, compared with commonly farmed large fish species (with SIS having a richer concentration of several important micronutrients)^(^ ^4^ ^)^. For this reason, our analysis compares and contrasts consumption according to the two categories: non-farmed and farmed fish.

The aims of the present paper are to describe the importance of fish in the diets of vulnerable groups in comparison to other ASF, and the contribution of species from non-farmed and farmed sources to nutrient intakes. We present quantitative food consumption data from a representative intra-household-level survey of rural Bangladesh, focusing on the first 1000 d of life (including pregnant women, lactating women and children less than 2 years of age). We describe differences in fish consumption among age groups, females and males, wealth groups and geographic regions. Our analysis reveals the relative importance of the two fisheries sub-sectors, capture fisheries and aquaculture, to vulnerable groups and can inform the research agenda for fisheries management, with the goal of optimising the nutritional, economic and environmental outcomes of the sector. In doing so we make an important contribution to the policy debate about how capture fisheries and aquaculture sub-sectors can best contribute to sustainable healthy diets^(^ ^7^ ^)^.

## Methods

Food consumption data from the Bangladesh Integrated Household Survey (BIHS) were analysed to describe fish consumption patterns, disaggregated by age and gender (in comparison to other ASF), wealth groups and geographic region^(^ ^10^ ^)^. The cross-sectional BIHS was conducted by the International Food Policy Research Institute, from October 2011 to March 2012, using a two-stage stratified sample design and was statistically representative of rural Bangladesh (77 % of the country’s population)^(^ ^11^ ^)^. The survey covered 5503 households, comprising 24198 individuals. Fish consumption from non-farmed and farmed sources was also compared across wealth groups and geographic regions. Households were categorised within wealth groups, based on per capita expenditure according to analysis of food and non-food expenditures. Group 1 represents the poorest 20 % and group 5 the wealthiest 20 % of surveyed households. In combination with data on nutrient composition of fish species, we used regression analyses to estimate nutrient intakes from fish from non-farmed and farmed sources.

### Dietary intake

The survey was comprehensive, covering many aspects of livelihoods and health status. Here, we used the modules on household food consumption (including quantitative data on raw ingredients used to prepare composite meals) and intra-household food consumption (including quantitative data on portions of cooked composite meals consumed by individuals within the household). We also used data from the modules on nutrition knowledge and awareness of mothers, and trial and adoption of sentinel practices. Data for both household and intra-household food consumption were collected from the person primarily responsible for meal preparation, using a 24 h recall method^(^ ^10^ ^)^. The intra-household data set provided only the cooked weight of composite foods (menu items) consumed by the individual (e.g. fish and vegetable curry); therefore calculation of the equivalent amount of raw ingredients consumed by each individual (to estimate nutrient intakes using food composition data) required the following calculation:

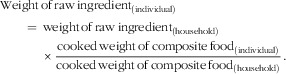




Quantities of dried foods (such as dried fish) were converted to raw weight equivalents using conversion factors based on the moisture content of foods (dried fish, factor of 3·5; fermented fish, factor of 2·2; dried meat, factor of 8·75^(^ ^12^ ^)^ (moisture content of fermented fish, 36 g moisture per 100 g; WorldFish, unpublished results).

### Procurement of fish

Knowledge of the ways in which fish is procured is useful in understanding interactions of different population groups with the market, own production of food, fishing activities and other ways of procuring fish. Data on the source of food procured at household level were collected from the person primarily responsible for food preparation (usually an adult female), using a 7 d recall. Sources of fish were recorded as: purchased, own production (e.g. in homestead ponds), collected, caught or fished, gifted, given as wages, loaned, begged or as sacrificial meat. In data presented here, ‘caught or fished’ and ‘collected’ were grouped together as these represent closely related activities. Fish sourced from a loan, begging or as sacrificial meat were grouped together as ‘other’ as they were reported in very low frequency. Only households which had procured fish in the last 7 d were included in this part of the analysis (*n* 5181).

### Source of fish production

Analysing the contributions of fish to nutrition according to production sub-sector (non-farmed or farmed) is pertinent for two reasons: (i) governance of the two sub-sectors is distinct and unique; and (ii) nutrient composition of species from these sub-sectors are distinct^(^ ^4^ ^)^. The sixty-three categories of fish recorded in the survey were grouped according to their primary production sub-sector (see online supplementary material, Table S1). Two local species, magur (*Clarias batrachus*) and shing (*Heteropneustes fossilis*), are commonly produced in both sub-sectors and so their contributions to fish consumption and nutrient intakes (see following section) were distributed evenly across both. All results for non-farmed fish consumption include dried fish (converted to fresh weight equivalent) and, in some cases, dried fish consumption is additionally presented as its own category.

### Nutrient intakes from fish

To estimate the nutrient intakes from fish, the raw weight of individual ingredients consumed by individuals was first adjusted by an average edible part conversion factor for small and large fish^(^ ^13^ ^)^, to account for parts such as large fish bones that are not consumed (see online supplementary material, Table S1). The adjusted individual portion was then multiplied by the nutrient composition of that species, based on published literature^(^ ^4^ ^,^ ^12^ ^,^ ^14^ ^)^. Some survey categories represented several species. In these cases, a simple weighted average nutrient composition was applied (Table S1). In the case of lack of data on nutrient composition of a species, expert opinion was sought on the most similar species (based on taxonomy) for which data were available (Dr MAR Hossain, Bangladesh Agricultural University, personal communication, 2015).

### Statistical analysis

Data analysis was conducted using the statistical software package Stata version 13.1. Individuals were excluded if they were currently residing away from the homestead, considered not a valid household member or they were a child being exclusively breast-fed and therefore not consuming other foods (individuals included, *n* 24 198). Regression analysis was used to estimate mean fish consumption while controlling for age (including a quadratic term), sex, wealth group and geographic region (*P*<0·05). In comparing nutrient intakes from fish from non-farmed and farmed categories, persons who did not consume any fish and persons who consumed fish from both categories on the one day surveyed were excluded (individuals included, *n* 14 525). In regression analysis, nutrient intakes from fish were the primary outcome variables (energy, protein, fat, Fe, Zn, Ca, vitamin A and vitamin B_12_), as predicted by category of fish (non-farmed or farmed), within the above specified model (*P*<0·05). All outcome variables were positively skewed in distribution. Repeat analysis on log-transformed variables revealed the same significance in results, except for protein (see ‘Results’ section). Due to a number of zero values for Zn, vitamin B_12_ and vitamin A intakes from fish (for species with nutrient composition not analysed, or analysed as having no detectable quantity of these nutrients), log transformation did not produce a normal distribution for those variables. In these cases, a sensitivity analysis was conducted using quantile regression which predicts the median rather than the mean and is therefore not influenced by skewness of the data. Energy intake was not adjusted for, as is commonly done in epidemiological studies^(^ ^15^ ^)^, because in this case energy intake from fish is considered an effect mediator rather than a confounder, given we modelled nutrient intakes as an outcome rather than as a predictor of disease risk. Sampling weights provided by the International Food Policy Research Institute derived from 2011 census data were applied in all results presented herein.

## Results

### Animal-source foods consumption

**Fig. 1:**
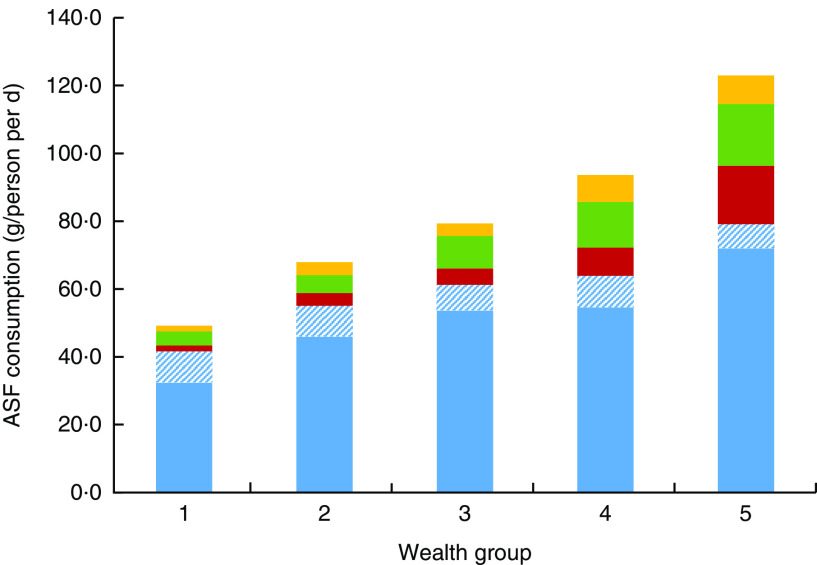
(colour online) Mean consumption (g/person per d) of animal-source foods (ASF; 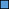, fresh fish; 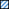, dried fish*; 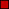, meat; 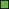, poultry; 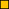, eggs) by wealth group† in rural Bangladesh (adjusted for age, sex and geographic region), October 2011–March 2012. *Dried fish includes fermented and dried fish, adjusted to fresh weight equivalent. †Wealth group 1 represents the poorest 20 % of households and wealth group 5 represents the wealthiest 20 % of households

Fish had by the far the highest average consumption (g/person per d) compared with other ASF (excluding milk, which was not analysed here) across all wealth groups in rural Bangladesh (see Fig. 1), with a mean national fish consumption of 60 (se 1) g/person per d, compared with 7 (se 1), 10 (se 1) and 5 (se <1) g/person per d for meat, poultry and eggs, respectively. Dried fish (adjusted to fresh weight equivalent) represented a considerable portion of total fish consumed, at 8 g/person per d, with no significant differences across wealth groups (*P*>0·05). Figure 1 shows that as a proportion of total ASF, fish was relatively more important for the poorest, constituting 85 % of mean total ASF consumption in the poorest group compared with 64 % of mean total ASF consumption in the wealthiest group.

### The first 1000 d

Consumption of ASF by pregnant women, lactating women and children less than 2 years of age followed a similar trend to national averages, with fish having by far the highest average consumption compared with other ASF (see Table 1). Total fish consumption of pregnant or lactating women was slightly higher than that of non-pregnant or non-lactating women of childbearing age. Mean fish consumption of lactating women in the current analysis is much higher than that reported by Yakes *et al*. (12·3 g/woman per d), the only other known quantitative data on fish consumption among lactating women in Bangladesh^(^ ^16^ ^)^. Consumption of meat, poultry and eggs by lactating women is also much higher than that reported by Yakes *et al*. (3·6 g meat and poultry/woman per d, 4·7 g eggs/woman per d)^(^ ^16^ ^)^. However, that was a very small study (259 women), carried out in only two locations in Northern Bangladesh, and so is not directly comparable.

**Table 1:** Mean consumption of animal-source foods (ASF; g/person per d) among children less than 2 years of age, pregnant women, lactating women and women of childbearing age in rural Bangladesh (adjusted for age, sex, wealth group and geographic region), October 2011–March 2012

		Total fish*		Dried fish†		Meat		Poultry		Eggs		
Group	*n*	Mean	se	Mean	se	Mean	se	Mean	se	Mean	se	Fish as % of all ASF
Children <2 years	726	7	1	1	<1	1	<1	1	<1	6	1	47
Pregnant women	262	69	5	10	2	10	4	13	4	4	1	73
Lactating women	1200	73	3	9	1	8	2	12	3	4	1	75
Women of childbearing age‡	5057	64	2	9	1	7	1	10	1	5	<1	74

* Total fish includes fresh, dried and fermented fish, converted to fresh weight equivalent.

† Dried fish includes dried and fermented fish, converted to fresh weight equivalent.

‡ Women aged 15–49 years, excluding pregnant or lactating women.

Understanding aspects of nutrition knowledge, attitudes and practices of caregivers is necessary for interpreting food consumption patterns within the first 1000 d of life. Regarding nutrition knowledge, when caregivers of children less than 2 years of age (*n* 979) were asked ‘What foods does a young child need in order to grow and develop their brain?’, fish was the highest-ranking primary response, reported by 29 % of women, followed by eggs, at 19 %. Regarding awareness, trial and adoption of feeding practices, when asked if they had heard about the practice of feeding ASF (such as fish, meat, liver or eggs) at least once daily to children older than 6 months, 78 % reported yes and 63 % reported that they had put this knowledge into practice. This is more optimistic when compared with actual consumption data showing that 44 % of children of complementary feeding age actually consumed ASF on the previous day (of which, 76 % consumed fish and the mean intake was 22 (se 1·6) g fish/child per d). For those who reported that they had not put this knowledge into practice, the most common reason (45 %) was that the child was not old enough, followed by financial limitations (39 %).

### Gender, age and wealth differences in fish consumption

Fish consumption was significantly lower for females than males from the age group 15 years or older (see Table 2). The largest discrepancies between females and males were within the 50 years or over age group, with females consuming 12 g/d less than males on average (*P*<0·001). There were no significant differences in fish consumption between females and males for infants, children and adolescents less than 15 years old. A similar pattern was found when examining gender differences in dried fish consumption (see Table 2). These findings are in contrast to previous studies, which although not of similar methods or sample size, have indicated that female children consume less ASF, including fish, than male children^(^ ^17^ ^,^ ^18^ ^)^.

**Table 2:** Mean total fish and dried fish consumption (g/person per d) by age and wealth group, overall and by gender, in rural Bangladesh (adjusted for age, sex, wealth group and geographic region), October 2011–March 2012

	Total fish*									Dried fish†				
	Overall		*n*		Female		Male			Female		Male		
	Mean	se	Female	Male	Mean	se	Mean	se	*P* value	Mean	se	Mean	se	*P* value
Age group (years)														
<2	7	1	368	358	6	1	7	1	0·61	1	1	2	<1	0·15
2–5	31	1	1143	1165	31	2	31	2	0·88	3	1	4	1	0·10
6–14	52	2	2730	2713	50	2	53	2	0·06	7	1	8	1	0·41
15–49	71	2	6483	5295	67	2	75	2	<0·001	9	1	11	1	0·002
≥50	67	2	1852	1961	61	2	73	2	<0·001	8	1	10	1	0·02
Wealth group‡														
1	42^a^	1	2620	2318	35	2	41	2	<0·001	8	1	10	1	0·01
2	55^b^	1	2551	2283	51	2	58	3	<0·001	8	1	9	1	0·06
3	61^c^	1	2447	2301	58	3	65	3	<0·001	7	1	8	1	0·03
4	64^c^	1	2489	2330	66	3	71	4	0·002	10	2	12	2	0·11
5	79^d^	1	2469	2260	78	3	83	4	0·07	7	1	7	1	0·84
Total	60	1	12 576	11 492	57	1	63	2	0·001	8	1	9	1	<0·001

^a,b,c,d^Mean values within a column with unlike superscript letters were significantly different (*P*<0·05).

* Total fish includes fresh, dried and fermented fish, converted to fresh weight equivalent.

† Dried fish includes dried and fermented fish, converted to fresh weight equivalent.

‡ Wealth group 1 represents the poorest 20 % of households and wealth group 5 represents the wealthiest 20 % of households.

When examining gender differences by wealth group, we found that total fish consumption by females was consistently and significantly lower than by males, for all wealth groups, except the wealthiest (Table 2). When examining dried fish, consumption by females was significantly lower than by males for wealth groups 1 and 3 (*P*<0·05). Of note is that there were no significant differences in mean consumption of dried fish (g/person per d) across wealth groups (*P*>0·05). However, dried fish as a proportion of total fish was more important for the poorer wealth groups, representing 21 % of total fish consumption (fresh weight equivalent), compared with 9 % in the wealthiest group. This trend is consistent with that observed by Belton *et al*. based on household data^(^ ^3^ ^)^. The importance of dried fish for the poor has been described elsewhere, based on surveys of varying sample sizes and methods; however, this is the first time it has been described at intra-household level.

### Procurement of fish

For the first time, data on procurement of fish consumed in rural Bangladesh is reported. The large majority of households (80 %) procured fish from a single source, most commonly through purchasing (71 % of households), followed by fishing-related activities (3 % of households) and own production (3 % of households). Only 20 % of households relied on procuring fish from a combination of two or more sources. Purchased fish accounted for 79–81 % of total fish consumed by wealth group, with the only significant difference between wealth groups 3 and 5 (Table 3, *P*<0·05). Reliance on fish from own production accounted for an average of 4–13 % of total fish consumption, with significant differences across wealth groups and geographic regions (*P*<0·05). By examining own production of fish *v*. fishing activities, we see that the poor relied more heavily on fishing activities (8 % of total fish consumed compared with 4 % from own production), whereas the rich relied much more heavily on own production (13 % of total compared with 4 % from fishing activities). The poor procured 8 % of total fish consumed as ‘gifted’ fish, which was significantly higher than the proportion of gifted fish consumed by all other wealth groups (*P*<0·05). When examining geographical differences in fish procurement, Khulna had the lowest reliance on purchased fish (73 % of total procurement) and the highest reliance on fish from own production. Rajshahi and Sylhet were the only regions in which the proportion of fish procured from fishing activities was higher than the proportion procured from own production. Fish procured from wages or other methods accounted for <1 % of total fish consumed and is not shown here.

**Table 3:** Proportion of fish consumption by procurement source (as a percentage of total fish consumed) according to wealth group and geographic division, rural Bangladesh, October 2011–March 2012

	Purchased*		Own production*		Fished/collected*		Gifted*	
	Mean	se	Mean	se	Mean	se	Mean	se
Wealth group†								
1	79^a,b^	2	4^a^	1	8^a^	1	8^a^	1
2	81^a,b^	1	5^b^	1	8^a,b^	1	5^b^	1
3	81^a^	1	6^b^	1	6^b^	1	5^b^	1
4	81^a,b^	1	8^c^	1	7^a,b^	1	4^b^	1
5	79^b^	2	13^d^	1	4^c^	1	4^b^	1
Geographic division‡								
Barisal	81^a,b^	3	8^a,b^	2	7^a,b^	2	4^a^	1
Chittagong	86^a^	2	5^a^	1	3^b^	<1	6^a^	1
Dhaka	80^b,c^	2	7^a^	1	6^a,c^	1	6^a^	1
Khulna	73^b,d^	4	13^b^	2	7^a^	1	6^a^	1
Rajshahi	81^a,c,d^	3	5^a^	1	7^a,b^	2	6^a^	1
Rangpur	83^a,c^	3	8^a,b^	2	4^b,c^	1	5^a^	1
Sylhet	80^a,c,d^	3	4^a^	1	11^a^	2	4^a^	1
Total	81	1	7	1	7	1	5	<1

^a,b,c,d^Mean values within a column with unlike superscript letters were significantly different (*P*<0·05), sections on wealth group and geographic division considered separately.

* All fish categories include dried and fermented fish, converted to fresh weight equivalent.

† Wealth group 1 represents the poorest 20 % of households and wealth group 5 represents the wealthiest 20 % of households. Means adjusted for geographic division.

‡ Means adjusted for wealth group.

### Consumption of non-farmed and farmed fish

**Fig. 2:**
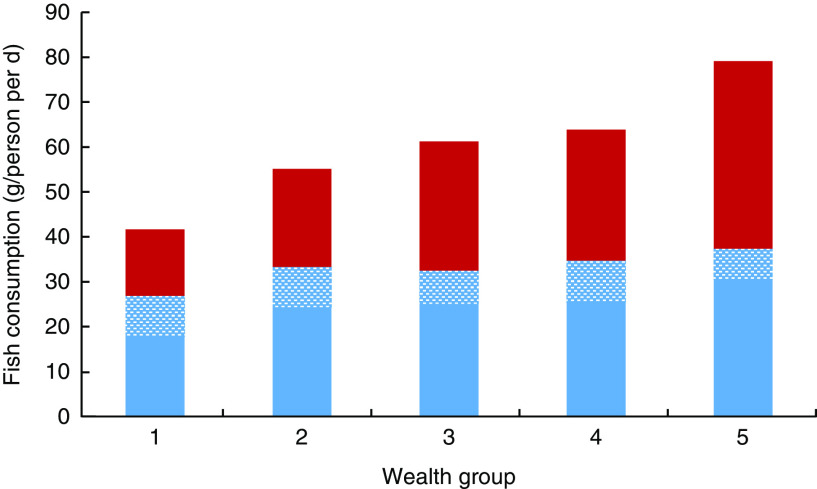
(colour online) Mean consumption (g/person per d) of non-farmed (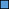, non-farmed fresh fish; 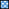, non-farmed dried fish*) and farmed fish (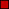) by wealth group† in rural Bangladesh (adjusted for age, sex and geographic region), October 2011–March 2012. *Dried fish includes fermented and dried fish, adjusted to fresh weight equivalent. The sum of non-farmed fresh fish and non-farmed dried fish equals total non-farmed fish consumption. †Wealth group 1 represents the poorest 20 % of households and wealth group 5 represents the wealthiest 20 % of households

When fish consumption was disaggregated according to the production source (non-farmed or farmed), non-farmed fish constituted the majority of consumption, with a national average of 33 g/person per d compared with 27 g/person per d from farmed fish (Table 4). This is dissimilar to previously reported results^(^ ^3^ ^)^. However, consumption of farmed fish species was likely to be over-represented in that analysis, as it was based on a survey covering four districts with a higher than average level of pond ownership and where participation in aquaculture-related development projects was common. Mean consumption of non-farmed fish ranged from 27 to 37 g/person per d across wealth groups, with consumption by the poorest wealth group significantly lower than by all other groups (*P*<0·05). In contrast, mean consumption of farmed fish increased significantly with each higher wealth group (with the only non-significant difference between wealth groups 3 and 4, *P*>0·05). In other words, consumption of non-farmed fish was relatively stable across wealth groups, but as wealth increased, consumption of fish from farmed sources increased. Figure 2 demonstrates that non-farmed fish (as a proportion of total fish consumption) was relatively more important in the diets of the poor, constituting 64 % of total fish consumption in the poorest wealth group compared with 47 % in the wealthiest group. This is consistent with the general trend reported by Belton *et al*.^(^ ^3^ ^)^.

**Table 4:** Mean fish consumption from non-farmed, farmed and dried fish (g/person per d) by geographic division in rural Bangladesh (adjusted for age, sex and wealth group), October 2011–March 2012

	Non-farmed fish*		Farmed fish		Dried fish†	
Division	Mean	se	Mean	se	Mean	se
Barisal	36^a^	3	21^a^	2	4^a,b^	1
Chittagong	37^a^	4	27^b^	2	14^c,d,e^	2
Dhaka	40^a^	2	34^c^	2	14^c,e^	2
Khulna	26^b^	3	35^c,d^	3	<1^f^	<1
Rajshahi	17^b^	3	28^b,d^	2	2^a^	1
Rangpur	24^b^	3	18^a^	2	9^b,d,e^	3
Sylhet	50^c^	3	29^b,c^	3	15^c^	2
Total	33	1	27	1	8	1

^a,b,c,d,e,f^Mean values within a column with unlike superscript letters were significantly different (*P*<0·05).

* Non-farmed fish includes fresh, dried and fermented fish, converted to fresh weight equivalent.

† Dried fish includes dried and fermented fish, converted to fresh weight equivalent.

### Geographic differences in fish consumption

Total fish consumption and the proportions from non-farmed and farmed sources varied by geographic region (Table 4). There were large variations in the total amount of fish consumed, with mean consumption in Sylhet (79 g/person per d) almost double that in Rangpur (42 g/person per d).

Consumption of non-farmed fish was significantly lower in Rajshahi, Rangpur and Khulna (17–26 g/person per d) compared with all other regions (36–50 g/person per d). Mean consumption of farmed fish by district was also highly variable (18–35 g/person per d), with consumption in Rangpur and Barisal significantly lower than in all other regions (*P*<0·05). The proportion of non-farmed to farmed fish also varied; non-farmed fish made up the majority of total fish consumed in all districts, except in Rajshahi and Khulna. These differences are likely due to varied availability and affordability of fish in different areas. Sylhet, for example, is a region with extensive inland wetlands and is therefore likely to have greater availability of non-farmed species. Khulna is a region with higher than average pond ownership, and also with commercial aquaculture farms, which most likely increase local fish availability. Dried fish consumption also varied considerably by geographic region, with mean consumption of 15 g/person per d (fresh weight equivalent) in Sylhet compared with <1 g/person per d in Khulna. In Chittagong, dried fish made up 22 % of total fish consumed, compared with 4 % in Rajshahi. This reflects a combination of geographical differences in both the availability of fish that are considered suitable for drying/fermenting and the cultural preferences that have hence evolved for such fish products.

### Nutrient intakes from fish

Analysing nutrient intakes from fish consumption according to fisheries sub-sectors is important, as there are large differences in nutritional quality of non-farmed and farmed fish species. Fish from non-farmed sources comprise a large variety of species from both inland and marine sources, many of which are SIS. These SIS when consumed whole, with head and bones, offer a particularly rich source of micronutrients such as Fe, Zn, Ca, vitamin A and vitamin B_12_. This is in contrast to farmed species, which are dominated by relatively few, large fish species, generally with lower micronutrient content^(^ ^4^ ^)^. This trend can be seen when comparing the mean nutrient intakes from fish (among those who consumed fish) from non-farmed and farmed categories, adjusted for age, sex, wealth group and geographic region (Table 5).

**Table 5:** Mean nutrient intakes (per person per d, among those who consumed fish) from non-farmed and farmed fish in rural Bangladesh (adjusted for age, sex, wealth group and geographic region), October 2011–March 2012

	Non-farmed fish*		Farmed fish			
Nutrient	Mean	se	Mean	se	*P* value	Daily RNI†
Energy (kJ)	284	8	336	9	<0·0001	10 800
Protein (g)	11·3	0·3	11·5	0·3	0·38§	47
Fat (g)	2·5	0·1	3·9	0·2	<0·0001	80
Fe (mg)	2·90	0·11	1·04	0·06	<0·0001	29·4
Zn (mg)‡	1·70	0·05	0·68	0·02	<0·0001	9
Ca (mg)	521	15	169	12	<0·0001	1000
Vitamin A (µg)‡	113	8	12	3	<0·0001	500
Vitamin B_12_ (µg)‡	1·77	0·08	1·44	0·08	<0·0001	2·4

RNI, recommended nutrient intake.

* Non-farmed fish includes fresh, dried and fermented fish, converted to fresh weight equivalent.

† Based on a female of reproductive age, with moderate physical activity level and weight of 57 kg. Fat requirements range from 25 to 35 % of total energy intake, which here is presented at the midpoint of the range (1 g fat=37 kJ).

‡ Using quantile regression, predicted mean Zn intake was 1·18 and 0·60 mg/person per d from non-farmed and farmed sources, respectively (*P*<0·0001); predicted mean vitamin B_12_ intake was 0·87 and 0·72 µg/person per d from non-farmed and farmed sources, respectively (*P*<0·0001); predicted mean vitamin A intake was 11 and 6 µg retinol activity equivalents/person per d from non-farmed and farmed sources, respectively (*P*<0·0001).

§ When repeated using log-transformed outcome variable, predicted mean differences in protein intake became statistically significant (*P*<0·0001).

Non-farmed fish made a larger contribution to nutrient intakes, for all micronutrients considered, than farmed fish (*P*<0·0001). There was no statistically significant difference in protein intakes between the two sub-sectors, and energy and fat intakes were both statistically significantly lower from non-farmed compared with farmed fish. Results are even starker when we consider the typical portion sizes of fish consumed from each sub-sector. Those who consumed farmed fish had approximately 18 g more fish than those who consumed non-farmed fish. So, even though the quantities of non-farmed fish consumed were smaller, the micronutrient contribution was greater than from farmed fish.

Statistically significant differences between nutrient intakes between the two sub-sectors were identified for all nutrients (except protein), but are these differences nutritionally important? In terms of energy, mean intake of farmed fish contributed 46 kJ more, per person per d; however, given that an active adult female’s typical estimated energy expenditure is about 10 000 kJ/d, a difference of <50 kJ is relatively unimportant (a difference of <0·5 % of the recommended nutrient intake (RNI) for an adult female). Similarly, the difference in fat intake from the two sub-sectors as a percentage of RNI is low (2 % of the RNI for an adult female). In contrast, when the differences in mean intakes of micronutrients (Fe, Zn, Ca, vitamin A and vitamin B_12_) between non-farmed and farmed fish are compared with their RNI, these differences are much more nutritionally important (a difference of 6–35 % of the RNI for an adult female). Both non-farmed and farmed fish make an equal and important contribution to protein requirements (approximately 25 % of daily RNI).

Differences in predicted intake of Zn, vitamin B_12_ and vitamin A between non-farmed and farmed fish were less stark when using quantile regression, which estimates the median intake rather than mean, but remained in the same direction and statistically significant (Table 5, *P*<0·001).

## Discussion

For the first time, using a national representative survey of rural Bangladesh, we have analysed quantitative data on ASF consumption, at the intra-household level, with focus on the first 1000 d of life. This analysis has shown significant variations in fish consumption related to wealth group, gender, age group and geographic location, all of which have implications for policies, programmes and interventions aiming to increase fish consumption and nutrient intake. Given that national nutrition guidelines recommend fish consumption of 60 g/person per d^(^ ^19^ ^)^, it is evident that targeted programmes to increase availability and affordability of fish for the people of the poorest two wealth groups nationally (whose consumption is much lower than this recommendation) should be prioritised. Fish consumption in females was significantly lower than in males, in all wealth groups except the wealthiest, and in those aged less than 15 years. Further investigation as to the nutritional significance of these gendered differences in fish consumption in relation to nutrient requirements is needed. For example, given that women generally have lower energy requirements than men, the lower consumption of fish may not be surprising; however, fish is a nutrient-rich food and its value lies more in provision of micronutrients rather than dietary energy. In this respect, particularly for women of childbearing age who often have higher nutrient requirements than men, these gendered differences in consumption are likely to be of nutritional significance. With respect to geographic trends, mean fish consumption in some regions, particularly Rangpur and Rajshahi, was much lower than the national recommendation, indicating that context-specific interventions to increase consumption are needed.

Consumption of dried fish formed a large component (9–29 %) of total fish consumption across age, gender and wealth groups, as well as in the first 1000 d of life. There were no differences in the quantity of dried fish consumed across wealth groups, although as a proportion of total fish, dried fish consumption was much more significant for the poor. Dried fish provides a concentrated source of micronutrients and potentially reduces the effects of seasonal availability of fresh fish (due to its longer shelf-life), and is therefore likely an important source of nutrients for particular groups. Given that nutrient-dense foods are desirable for young children (given their high nutrient needs and low stomach volume capacity)^(^ ^20^ ^)^, dried fish may be well suited to the dietary needs of infants and young children. Further research into the dried fish value chain, including food safety aspects, seasonality of consumption and nutritional quality of dried fish, is necessary.

In relation to fish consumption in the first 1000 d of life, it is evident that the predominant reason for not feeding fish to infants from 6 months of age was due to the perception that they are too young, even though caregivers knew that fish is a nutritious food recommended for young children. One plausible explanation identified elsewhere is that caregivers are concerned about suitability of fish with regard to consistency (e.g. presence of bones) for young children^(^ ^21^ ^)^. This represents an opportunity for education and training on optimal complementary feeding practices, including modified preparation methods for fish particularly suited to infants. Innovative food preparation methods such as fish-based food products, using powdered dried fish designed specifically for this age group, may be an appropriate alternative^(^ ^22^ ^)^. Overall, mean fish intakes among pregnant and lactating women were surprisingly slightly higher than the national average. Maternal seafood consumption throughout pregnancy has been shown to improve neurodevelopmental outcomes of infants and children^(^ ^23^ ^,^ ^24^ ^)^. Breast milk and fish have been identified as the most important dietary sources of *n*-3 fatty acids for infants of complementary feeding age. Furthermore, consumption of marine foods by the mother appears to be the most important determinant of *n*-3 fatty acids content of breast milk^(^ ^25^ ^)^. Therefore, further investigation of the enablers of and barriers to increased fish consumption, particularly for women of childbearing age, is necessary.

Purchased fish formed the majority of total fish consumed for households across all wealth groups and locations. However, there were important differences in fish procurement among wealth groups. The poor were more reliant on fishing activities (capture fisheries) and the rich more on own production (aquaculture).

When considering the source of fish production, we have shown that non-farmed fish makes a greater contribution to micronutrient intakes compared with farmed fish in rural Bangladesh. This is likely due to the large diversity of species within the non-farmed fish category, each with a unique nutrient profile, as well as differences in the edible portion of SIS which make up a large proportion of the non-farmed category compared with farmed large fish. SIS are commonly consumed whole, with head, bones and sometimes viscera, which contributes to the nutritional diversity of fish as consumed, compared with commonly farmed species which are typically large in size and only the flesh is consumed^(^ ^26^ ^)^. The differences in micronutrient content between commonly farmed large species and selected common SIS are large; in many cases, more than an order of magnitude^(^ ^4^ ^)^. While there is no question that aquaculture has an important role to play in increasing fish availability and stabilising prices^(^ ^27^ ^)^, policies and production technologies that enhance access to and consumption of SIS (compared with large, commonly farmed species) by vulnerable groups are likely to have greater impacts on nutrition (given their higher micronutrient content, on a direct substitution basis). Economic analysis was considered outside the scope of the present study; however, other work has demonstrated the lower price and income elasticity of SIS compared with large fish, particularly for the poor^(^ ^28^ ^)^. This indicates that: (i) SIS are considered more of a staple food (rather than a luxury) compared with large fish; and (ii) changes in the price of large fish will bring about larger changes in consumption (either increases or decreases).

There are several initiatives in Bangladesh that show great potential to enhance consumption of SIS, but they are yet to receive adequate policy attention and be widely disseminated. For example, WorldFish and partners have developed a polyculture technology in which the micronutrient-rich SIS, mola (*Amblypharyngodon mola*), is produced in homestead ponds along with farmed large-sized species such as carp^(^ ^29^ ^)^. The SIS breed in the pond and are ideally suited for regular harvesting and consumption by the household, whereas the large fish, stocked as fingerlings, are harvested as adults and are suited for sale. This technology has been shown to be a cost-effective approach to the reduction of micronutrient malnutrition^(^ ^30^ ^)^. This research should be further developed to incorporate other SIS. Another example of a promising production technology is enhanced stocking of SIS in wetlands (as an alternative to the well-documented method of stocking exotic or indigenous large species), with benefits for increased productivity, sustainability, biodiversity and nutrition^(^ ^31^ ^)^. Other authors have recognised the underutilised but auspicious practice of rice–fish production systems, another diverse production system traditional to many Asian countries but largely forgotten during the Green Revolution focus on rice production^(^ ^32^ ^)^.

The important policy and programme implications of the detailed analysis presented here demonstrate the value of intra-household-level data, as well as consumption surveys in which foods are recorded at species/variety level. Investment in methods and tools for collection of high-quality food consumption data should be prioritised in other countries where fish are an important ASF. High-quality and detailed data will inform and maximise the potential of policies and programmes designed to improve diets and nutrition, particularly among vulnerable groups.

Limitations of the study relate largely to the survey design. The BIHS was nationally representative of rural Bangladesh, and therefore the results are not generalisable to the urban population. Furthermore, the survey was conducted over a period of 6 months. Food insecurity is known to vary seasonally and spatially, reflecting diverse production and harvest systems across the country^(^ ^33^ ^)^. Data collected periodically throughout the year are required to understand seasonal differences in fish consumption within geographic regions, particularly among vulnerable groups.

## Conclusion

The present research confirms that policies and programmes aimed to increase fish consumption as a means to improve nutrition in rural Bangladesh should focus on women and young children, on households from the poorest wealth groups, and particularly those in Rajshahi and Rangpur. Aquaculture will continue to play an important role in increasing the availability and affordability of fish; however, fish species from the non-farmed sector are better placed to contribute to greater micronutrient intakes, on a direct substitution basis. This presents an opportunity for aquaculture to contribute to improved nutrition through development and scale up of diverse production technologies suitable for different agro-ecologies and producing diverse fish species, including SIS. There are several good examples of diverse production systems (such as homestead pond polyculture systems with SIS, rice–fish systems and stocking of wetlands with SIS) which show potential for increasing availability of this nutritious food. Protection and promotion of sustainable capture fisheries remain imperative, and should be considered as complementary to aquaculture development. Details of the nutritional quality and safety of fish species remain a significant gap and research in this field must be given high priority. Our findings are of significance to many other regions where small fish are commonly consumed (as fresh, dried or processed in condiments), such as South East Asia, the African Great Lakes region and Latin America.

## Supplementary material

For supplementary material accompanying this paper visit https://doi.org/10.1017/S1368980016002615. click here to view supplementary material
