# Supplementary material for: Non-farmed fish contribute to greater micronutrient intakes than farmed fish: results from an intra-household survey in rural Bangladesh
Source: Public Health Nutr. 2016 Oct 5;20(4):702–11. doi: 10.1017/S1368980016002615 (PMC5468797; doi:10.1017/S1368980016002615)
Supplement: Supplementary file 1 [file S1368980016002615sup001.docx]

# Supplementary material

Table S1: Survey categories of fish consumption grouped according to primary production sub-sector, and matched to nutrient composition data*

| **BIHS Survey Code** | **BIHS Survey Fish Category** | **Scientific name used for  nutrient composition** | **Edible parts conversion factor** | | **Nutrient composition per 100 g raw, edible parts** | | | | | | | |
| --- | --- | --- | --- | --- | --- | --- | --- | --- | --- | --- | --- | --- |
|  |  |  |  | | **Energy (kJ)** | **Protein (g)** | **Fat (g)** | **Iron (mg)** | **Zinc (mg)** | **Calcium (mg)** | **Vitamin B12 (µg)** | **Vitamin A (µg RAE)** |
|  | Non-farmed fish |  | |  |  |  |  |  |  |  |  |  |
| 180 | Surma | *Scomberomorus guttatus*^(12)^ | | 0.830 | 431 | 20.9 | 2.0 | 0.80 | 0.78 | 35.0 | NA | NA |
| 181 | Chital | *Chitala chitala*^(12)^ | | 0.830 | 405 | 17.8 | 2.8 | 1.60 | 0.61 | 104.0 | NA | 30 |
| 182 | Boal | *Wallago attu*^(12)^ | | 0.830 | 339 | 15.4 | 2.1 | 0.80 | 0.27 | 83.0 | NA | 1 |
| 183 | Aair | *Sperata aor*^(12)^ | | 0.830 | 373 | 17.0 | 2.3 | 0.90 | 0.23 | 11.0 | NA | NA |
| 185 | Ritha | *Arius truncatus* ^†(14)^ | | 0.830 | 385 | 18.7 | 1.9 | 4.20 | NA | 98.0 | NA | NA |
| 186 | Hilsa | *Tenualosa ilisha* | | 0.830 | 1020 | 16.4 | 18.3 | 1.90 | 1.20 | 220.0 | 2.30 | 20 |
| 187 | Jatka | *Tenualosa ilisha (jatka)* | | 0.830 | 618 | 19.0 | 7.7 | 2.50 | 1.80 | 500.0 | 2.00 | 14 |
| 193 | Shole/gozar | *Channa striatus, Channa marulius* | | 0.830 | 298 | 17.9 | 0.3 | 0.42 | 0.67 | 53.0 | 0.88 | 0 |
| 194 | Taki | *Channa punctatus* | | 0.867 | 306 | 18.3 | 0.6 | 1.80 | 1.50 | 766.0 | 1.60 | 139 |
| 197 | Baim | *Mastacembelus armatus* | | 0.867 | 381 | 17.9 | 1.7 | 1.90 | 1.10 | 449.0 | 1.72 | 27 |
| 199 | Meni | *Nandus nandus* | | 0.867 | 338 | 16.7 | 1.7 | 0.84 | 1.60 | 1300.0 | 0.90 | 60 |
| 200 | Shapla/padda/rupsha | *Dasyatis kuhlii, D. uarnak, D. zugei*^(14)^ | | 0.830 | 368 | 21.3 | 0.3 | 0.60 | NA | 18.0 | NA | 11 |
| 205 | Large dried fish | *Lates calcarifer* ^‡(12)^ | | 1.0 | 1340 | 60.2 | 8.6 | 3.00 | 0.57 | 939.0 | NA | NA |
| 211 | Gura mach | Mixed small fish ^§^ | | 0.867 | 410 | 15.6 | 4.0 | 4.46 | 3.38 | 1085.0 | 6.87 | 587 |
| 212 | Panch mishali | Mixed small fish ^§^ | | 0.867 | 410 | 15.6 | 4.0 | 4.46 | 3.38 | 1085.0 | 6.87 | 587 |
| 213 | Puti | *Puntius sophore / Puntius ticto* | | 0.867 | 463 | 15.6 | 5.3 | 2.80 | 3.35 | 1261.0 | 5.38 | 38 |
| 214 | Tengra | *Mystus vittatus* | | 0.867 | 428 | 15.1 | 4.6 | 4.00 | 3.10 | 1093.0 | 3.50 | 12 |
| 215 | Pabda | *Ompok pabda* | | 0.867 | 619 | 16.2 | 9.5 | 0.46 | 0.90 | 91.0 | NA | NA |
| 216 | Moa/mola | *Amblypharyngodon mola* | | 0.867 | 445 | 17.3 | 4.5 | 5.70 | 3.20 | 853.0 | 7.98 | 2503 |
| 217 | Dhela | *Osteobrama cotio cotio* | | 0.867 | 387 | 14.7 | 3.8 | 1.80 | 3.70 | 1200.0 | 4.70 | 918 |
| 218 | Batashi | *Ailia coila* | | 0.867 | 751 | 17.1 | 12.6 | 0.82 | 1.20 | 110.0 | 4.10 | 37 |
| 219 | Kachki | *Corica soborna* | | 0.867 | 267 | 11.9 | 1.9 | 2.80 | 3.10 | 476.0 | 3.55 | 78 |
| 220 | Chanda | *Pseudambassis ranga* | | 0.867 | 400 | 15.5 | 3.8 | 2.10 | 2.60 | 1153.0 | 6.42 | 336 |
| 221 | Khalisa | *Colisa fasciata* | | 0.867 | 354 | 15.2 | 2.5 | 4.10 | 2.30 | 1700.0 | 5.55 | 46 |
| 222 | Chela | *Chela cachius* | | 0.867 | 349 | 15.2 | 2.4 | 0.84 | 4.70 | 1000.0 | 5.64 | 132 |
| 223 | Chapila | *Gudusia chapra* | | 0.867 | 385 | 15.5 | 3.8 | 7.60 | 2.10 | 1063.0 | 6.99 | 73 |
| 224 | Kajari | *Ailia coila* | | 0.867 | 751 | 17.1 | 12.6 | 0.82 | 1.20 | 110.0 | 4.10 | 37 |
| 225 | Tatkeni | *Crossocheilus latiusa* ^†(12)^ | | 0.867 | 405 | 15.3 | 3.9 | 2.20 | 1.09 | 195.0 | NA | NA |
| 226 | Bata | *Labeo bata*^(12)^ | | 0.867 | 446 | 15.9 | 4.7 | 1.20 | 0.94 | 493.0 | NA | NA |
| 227 | Ghutum | *Lepidocephalichthys guntea* | | 0.867 | 431 | 17.2 | 3.9 | 3.30 | 2.50 | 950.0 | 8.75 | 76 |
| 228 | Bele | *Glossogobius giuris* | | 0.867 | 292 | 16.6 | 0.4 | 2.30 | 2.10 | 790.0 | 2.10 | 18 |
| 229 | Chewa | *Glossogobius giuris* | | 0.867 | 292 | 16.6 | 0.4 | 2.30 | 2.10 | 790.0 | 2.10 | 18 |
| 230 | Poa | *Protonibea diacanthus*^(12)^ | | 0.867 | 422 | 18.6 | 2.9 | 0.40 | 0.65 | 32.0 | NA | 17 |
| 231 | Foli | *Notopterus notopterus* | | 0.867 | 384 | 20.5 | 0.6 | 1.70 | 1.60 | 230.0 | 2.00 | 0 |
| 232 | Bacha | *Eutrpiichthys vacha*^(12)^ | | 0.867 | 512 | 16.1 | 6.4 | 0.70 | NA | 520.0 | NA | NA |
| 233 | Baicha | *Colisa fasciata* ^†^ | | 0.867 | 354 | 15.2 | 2.5 | 4.10 | 2.30 | 1700.0 | 5.55 | 46 |
| 234 | Kaikla | *Xenontedon cancila* | | 0.867 | 329 | 17.1 | 1.2 | 0.65 | 1.90 | 610.0 | 2.89 | 91 |
| 235 | Darkini | *Esomus danricus* | | 0.867 | 384 | 15.5 | 3.2 | 12.00 | 4.00 | 891.0 | 12.50 | 660 |
| 236 | Palshe | *Liza parsia* | | 0.867 | 813 | 16.1 | 14.3 | 1.30 | 0.84 | 66.0 | NA | NA |
| 239 | Kakra | *Scylla serrata*^(14)^ | | 0.830 | 411 | 17.9 | 2.9 | 2.60 | NA | 183.0 | NA | 218 |
| 240 | Small prawn | *Macrobrachium malcolmsonii* | | 0.867 | 364 | 15.7 | 2.2 | 13.00 | 3.30 | 1200.0 | NA | NA |
| 241 | Dried small shrimp/prawn | *Penaeus sp. Palaemon spp* ^‡(14)^ | | 1.0 | 1322 | 69.1 | 4.0 | 5.30 | 3.20 | 770.0 | NA | 40 |
| 242 | Dried small fish | *Puntius sophore, Setipinna phasa* ^‡^ ^¶^ ^(12)^ | | 1.0 | 1610 | 62.0 | 14.7 | 25.00 | 12.00 | 2540.0 | NA | 8 |
| 243 | Fermented fish | *Puntius sophore, Setipinna phasa* ^║¶^ | | 1.0 | 1049 | 37.6 | 10.1 | 20.98 | 4.22 | 432.0 | NA | NA |
| 908 | Other big fish | Average of survey codes 176-193^(12; 14)^ | | 0.830 | 473 | 17.48 | 4.7 | 1.34 | 1.05 | 166.0 | 2.00 | 32 |
| 909 | Other small fish | Mixed small fish ^§^ | | 0.867 | 410 | 15.6 | 4.0 | 4.46 | 3.38 | 1085.0 | 6.87 | 587 |
|  | Farmed fish |  | |  |  |  |  |  |  |  |  |  |
| 176 | Rui | *Labeo rohita* | | 0.830 | 422 | 18.2 | 3.0 | 0.98 | 1.00 | 51.0 | 5.05 | 13 |
| 177 | Katla | *Catla catla* | | 0.830 | 267 | 14.9 | 0.7 | 0.83 | 1.10 | 210.0 | 1.30 | 22 |
| 178 | Mrigel | *Cirrhinus mrigala* | | 0.830 | 363 | 18.9 | 1.1 | 2.50 | 1.50 | 960.0 | 5.57 | 15 |
| 179 | Kalibaus | *Labeo calbasu* ^(12)^ | | 0.830 | 400 | 17.0 | 3.0 | 1.10 | 0.36 | 13.0 | NA | NA |
| 184 | Pangash | *Pangasianodon hypophthalmus* | | 0.830 | 925 | 16.0 | 17.7 | 0.69 | 0.65 | 9.0 | 1.50 | 31 |
| 188 | Grass carp | *Ctenopharyngodon idella* | | 0.830 | 341 | 15.2 | 1.1 | 0.46 | 0.91 | 54.0 | NA | NA |
| 189 | Mirror carp | *Cyprinus carpio* | | 0.830 | 381 | 16.4 | 2.9 | 1.10 | 2.20 | 37.0 | NA | 2 |
| 190 | Silver carp | *Hypophthalmichthys molitrix* | | 0.830 | 435 | 17.2 | 4.1 | 4.40 | 1.40 | 903.0 | 0.55 | 0 |
| 191 | Telapia | *Oreochromis niloticus* | | 0.830 | 390 | 19.5 | 2.0 | 1.10 | 1.20 | 95.0 | 0.70 | 10 |
| 192 | Swarputi | *Barbonymus gonionotus* | | 0.830 | 729 | 17.4 | 11.7 | 0.60 | 0.74 | 227.0 | NA | NA |
| 198 | Koi | *Anabas testudineus* | | 0.867 | 737 | 15.5 | 12.8 | 0.87 | 0.60 | 85.0 | 2.38 | 295 |
| 201 | Bagda chingree | *Penaeus monodon*^(12)^ | | 0.830 | 388 | 16.5 | 2.9 | 0.60 | 1.73 | 17.0 | NA | NA |
| 202 | Golda chingree | *Macrobrachium rosenbergii*^(12)^ | | 0.830 | 431 | 20.9 | 2.0 | 0.70 | 1.25 | 18.0 | NA | 2 |
| 204 | Poona fish | *Oreochromis niloticus* | | 0.876 | 412 | 19.0 | 2.6 | 0.69 | 0.65 | 9.0 | 2.50 | 21 |
| 238 | Karfu fish | *Cyprinus carpio* | | 0.830 | 381 | 16.4 | 2.9 | 1.10 | 2.20 | 37.0 | NA | NA |
|  | Fish both non-farmed and farmed | | |  |  |  |  |  |  |  |  |  |
| 195 | Magur | *Clarias batrachus* | | 0.867 | 326 | 16.5 | 1.3 | 1.20 | 0.74 | 59.0 | 4.83 | 25 |
| 196 | Singi | *Heteropneustes fossilis* | | 0.867 | 374 | 19.1 | 1.9 | 2.20 | 1.10 | 60.0 | 12.80 | 32 |

BIHS, Bangladesh Integrated Household Survey; RAE, retinol activity equivalent; NA, Data not available.

*Nutrient composition data are sourced from Bogard et al (2015)^(4)^, except where specified otherwise by citation in ‘Scientific name used for nutrient composition’ column.

† No composition data was available for exact species, so composition data from a similar fish from the same family was used.

‡ Conversion factor of 3.5 used to estimate fresh weight equivalent.

§ Average of *Pseudambassis ranga, Gudusia chapra, Chela cachius, Esomus danricus, Osteobrama cotio cotio, Puntius sophore, Puntius ticto, Amblypharyngodon mola*.

║ Conversion factor of 2.2 used to estimate fresh weight equivalent.

*¶*Nutrient composition sourced from WorldFish unpublished data.
